# History of Medical Education and Institutions in the United States, Etc.

**Published:** 1850-11

**Authors:** 


### ARTICLE IV.

*History of Medical Education and Institutions in the United States, from the first settlement of the British Colonies to the year* 1850, *with a chapter on the present condition and wants of the Profession, and the means necessary for supplying those wants and elevating the character, and extending the usefulness of the whole Profession.*—By N. S. Davis, M.D., Professor of Principles and Practice of Medicine in Rush Medical College; member of the American Medical Association; Permanent member of the Medical Society of the State of New York; corresponding member of the N. Y. Medical Association; member of the Illinois State Medical Society, etc. pp. 228; 12mo. Chicago: S. C. Griggs & Co., 1850. (From the Publishers.)

Our readers will, with this number of the Journal, receive the conclusion of a copy of this valuable work, and of course it will be unnecessary for us to give an analysis of its interesting contents. We congratulate the physicians of the whole country upon the privilege, now for the first time offered them, of reading a connected history of their profession in this country. It should be in every American physician's library; for who, that loves his profession, does not wish to learn its history in his own country.

The neat and substantial manner in which this, the first medical book published in our city, is got up, speaks well for the publishers, and adds another to the numerous evidences of western skill and enterprise.

